# Switzerland’s Dependence on a Diamorphine Monopoly

**DOI:** 10.3389/fpsyt.2022.882299

**Published:** 2022-05-09

**Authors:** Caroline Schmitt-Koopmann, Carole-Anne Baud, Valérie Junod, Olivier Simon

**Affiliations:** ^1^Service of Addiction Medicine, Lausanne University Hospital (CHUV), University of Lausanne, Lausanne, Switzerland; ^2^Faculty of Business and Economics, University of Lausanne, Lausanne, Switzerland; ^3^Faculty of Law, University of Geneva, Geneve, Switzerland

**Keywords:** diamorphine, Switzerland, market, manufacturing, shortages

## Abstract

In 2021, the manufacturer of diamorphine reported a possible impending shortage for Switzerland and Germany. This led us to investigate this controlled medicine’s manufacture, market, and regulatory constraints. Based on our analysis of legal texts and gray literature in the form of reports and documents, we propose recommendations to prevent and address diamorphine shortages in Switzerland. Diamorphine, also known as pharmaceutical “heroin,” is used medically to treat persons with severe opioid use disorder in a handful of countries. The controlled medicine is manufactured from morphine, which, in turn, is extracted from opium poppies. Studying data from the International Narcotics Control Board for 2019, we find that Switzerland accounts for almost half of the worldwide medical consumption of diamorphine. It manufactures more than half of the worldwide total and keeps the largest stocks. Moreover, Switzerland is dependent on a sole supplier of diamorphine (monopoly). As a niche product, diamorphine has an increased risk of shortage. Such a shortage would immediately threaten a valuable public health program for around 1,660 Swiss patients. We believe it is urgent to curtail the monopoly and ensure a stable supply for the future.

## Introduction

Diamorphine, also known under its old brand name Heroin, is one of the most controversial substances worldwide. According to the 1961 Single Convention, it is a schedule I drug, meaning it is subject to the highest degree of control (1). In Switzerland, diamorphine is classified as a prohibited controlled substance (schedule d), but it can be used as a medicine under particular conditions (2).

Indeed, the Swiss Agency for Therapeutic Products (Swissmedic) approved diamorphine (under the brand name Diaphin) for treating persons with severe opioid use disorder in 2001 (3). Treatment with diamorphine prescription (TDP) is the only approved effective alternative for persons who do not respond to classic opioid agonist treatments (OAT), such as methadone or buprenorphine treatment (4, 5). It was introduced in 1994 in Switzerland as a pragmatic solution to an AIDS crisis and open drug scenes (6). Under heavy criticism at first, it is now enshrined in Swiss law (6) and recognized internationally as a successful and cost-effective public health program (5, 7, 8). In 2019, 1,663 persons in Switzerland received TDP, which has been reimbursed since 2002 by health insurers (6, 9).

In Switzerland, DiaMo Narcotics GmbH holds the marketing authorization for the only three diamorphine products available: Diaphin i.v. (intravenous), Diaphin IR (immediate-release) tablets, and Diaphin SR (slow-release) tablets (3). DiaMo Narcotics GmbH sources the active ingredient, diamorphine, from two active ingredient manufacturers (10). Two other contract manufacturers then formulate the active ingredient: One contract manufacturer formulates Diaphin i.v., whereas Diaphin IR and SR tablets are formulated by another (10). Due to the small order volume, finding another contract manufacturer is not a realistic option, as noted by a report which analyzed the diamorphine supply situation in January 2021 (10).

In theory, other opioid products could constitute alternatives to diamorphine, but none so far have been authorized for this indication.

### The 2021 Diamorphine Supply Disruption

The risk of a diamorphine shortage was already identified in 2016 by the Swiss Federal Council (11). In November 2020 the risk of a supply shortage of Diaphin i.v. became acute, due to the bankruptcy of the contract manufacturer, which formulated Diaphin i.v.: Legacy Pharmaceuticals (10). There were no stocks of Diaphin i.v. because of past difficulties with the contract manufacturer (10).

The impending shortage was not made public, and communication was restricted to the involved Swiss authorities and TDP centers. According to a report on the Swiss TDP, the communication regarding the supply was not transparent enough (10). In contrast to Swiss authorities, the German Medicines Agency announced an impending supply disruption of Diaphin i.v. for the German market in March 2021 (12). Based on publicly available information it is not possible to know if the bankruptcy of Legacy Pharmaceuticals also caused the German Diaphin i.v. supply disruption.

Shortages of diamorphine pose a significant problem for people in TDP and their treating physicians. Risks associated with treatment discontinuation are poorer mental and physical health, increased illegal activities, and consumption of illicit substances (13). Hence, a rapid solution is needed to supply the affected population in case of a shortage.

### Structure of the Article

This article describes Switzerland’s dependence on a diamorphine monopoly. We analyzed legal texts and gray literature in the form of reports and documents from Swiss and other authorities. Furthermore, we used reports and data from the International Narcotics Control Board (INCB).

Section “How Did Diamorphine Become a Niche Product?” will describe how the once widely used diamorphine has become a highly regulated niche product. The manufacturing and diamorphine market will be analyzed in sections “How Is Diamorphine Manufactured?” and “How Is the Market for Diamorphine Structured?” In section “Are There Legal or Regulatory Constraints Preventing the Introduction of a Diamorphine Generic in Switzerland?” we identify relevant regulations for diamorphine manufacturers in Switzerland. In section “How Did Switzerland Address the 2021 Supply Disruption?” we analyze the response to the 2021 supply disruption in Switzerland and compare it to the United Kingdom (UK). Lastly, we formulate actionable recommendations in section “What Do We Recommend?”

This research is part of a project funded by the Swiss National Science Foundation (SNSF grant number 182477), which aims to analyze the current controlled medicines legislation in Switzerland.

## How Did Diamorphine Become a Niche Product?

Under the brand name heroin, Bayer started commercial production of pharmaceutical diamorphine in 1898; a few years later, other pharmaceutical companies started offering diamorphine as well (14). In the late nineteenth century and early twentieth century, diamorphine was widely used and marketed for “heavy coughs, to relieve the pain of childbirth and serious war injuries, prepare patients for anesthesia, and control certain mental disorders” (15). At the turn of the century, addiction caused by diamorphine was first recognized as a problem; the first so-called “morphine maintenance clinics” were set up to treat persons with opioid use disorder (16, 17).

Internationally, the 1912 International Opium Convention aimed to control opiates strictly but was not implemented globally due to the outbreak of World War I (17). Diamorphine production was limited after the 1931 Geneva Convention established constraints and many countries banned the substance (16, 17). In 1961, the Single Convention on Narcotic Drugs replaced previous treaties and is in its revised version still in force today (1, 18).

At a national level, the United States (US) passed the Harrison Act in 1914, introducing federal narcotics controls (14, 16, 19), followed by an outright ban on diamorphine in 1924 (16, 19).

In contrast, in the United Kingdom (UK), the 1926 Rolleston report laid the groundwork to continue administering morphine or diamorphine to treat persons with an opioid (heroin) use disorder who did not respond to abstinence-based programs (20, 21). The so-called “British system” remained intact until the passing of the Dangerous Drugs Act in 1967, which restricted the right to prescribe diamorphine to specifically licensed doctors only (22). This led to a decrease in people treated with diamorphine (23–25). Clinical guidelines published in 1984 cemented the shift from injectable diamorphine to oral methadone prescription (22). Interestingly, the UK remains the only country in the world to also use diamorphine for severe pain associated with surgical procedures, myocardial infarction, or pain in the terminally ill, and for the relief of dyspnea in acute pulmonary edema (26).

Switzerland passed its first narcotics law that introduced an authorization requirement for manufacturing and trading opiates and cocaine in 1924 (27). Still in force today is the Federal Act on Narcotics and Psychotropic Substances (NarcA), which was passed in 1951 (28). It has often been revised, with the last major change dating to 2011 when the four-pillar policy (prevention, therapy, harm reduction, repression) and TDP were enshrined in the law. Aside from Switzerland, TDP is currently only available in Denmark, Germany, Luxembourg, the Netherlands, the United Kingdom, and Canada (29).

In conclusion, after being widely used, diamorphine is nowadays a niche product in few countries with low production volumes, putting it at high risk of shortage.

## How Is Diamorphine Manufactured?

The licit manufacture of diamorphine starts with the cultivation of poppy plants (Papaver somniferum) on licensed fields (30). Papaver somniferum contains, among other alkaloids, morphine, codeine, and thebaine (31). All three are chemically related and easily convertible into one another (31). Breeding allowed for the creation of varieties that yield higher amounts of morphine or other opioid alkaloids (31).

**FIGURE 1:**
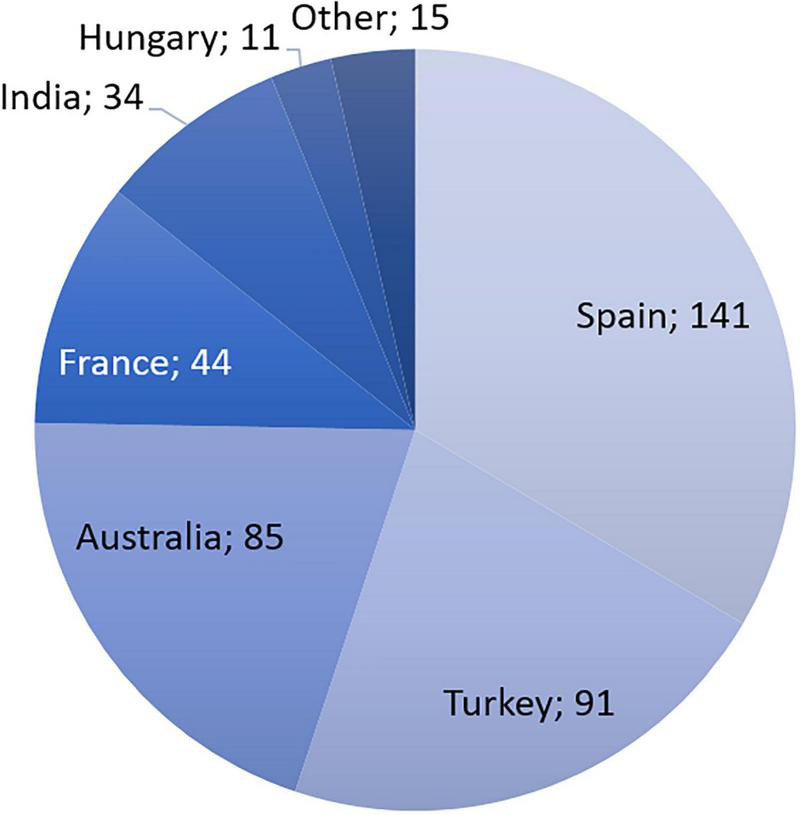
Production of opiate raw materials rich in morphine in tons of morphine equivalent, 2019. Based on data from INCB technical report 2020.

According to the INCB, the main licit cultivation countries are Australia, France, Hungary, India, Slovakia, Spain, and Turkey (Figure 1) (32). In 2019, Spain was the largest producer of morphine-rich raw materials (141 metric tons), followed by Turkey (85 metric tons), Australia (85 metric tons), and France (44 metric tons). Taken together, these four countries were estimated to account for 91% of the licit global production of morphine-rich raw materials in 2020.

The fully matured poppy plants’ field-dried leaves, stalks, and seedpods are used to make poppy straw concentrate (1). In the next step, morphine is extracted from the poppy straw concentrate before reacting with either acetic acid or acetic anhydride to produce diamorphine. Pure diamorphine, both as a base and as a hydrochloride salt, is a colorless crystalline solid (33). All currently available diamorphine preparations for injection are lyophilized powders because an aqueous solution is not chemically stable enough for storage (34).

Whilst opiates are still manufactured from the opium poppy, advances in synthetic biology enable the creation of a yeast strain capable of making morphine from glucose (35). Four groups of researchers introduced different genetic components into the yeast genome, which combined constitute the entire morphine synthesis pathway (36–41). A morphine manufacturing yeast strain could change the current supply chain, as the manufacture of morphine by self-replicating yeast might be cheaper and more stable than the current processes. However, to the best of our knowledge, no morphine product using this technology has entered the market.

Consequently, the current dependence on the cultivation of Papaver somniferum is a risk factor that could lead to supply disruptions for morphine and diamorphine. The available product on the global market is subject to yearly fluctuations, for example, due to heavy rains or droughts (42). Additionally, the overall demand for opioids is increasing because developing nations require appropriate amounts of opioids to treat pain. For example, in Uganda, opioid analgesic consumption increased by 342% from 2000 to 2015 but overall remained extremely low (43). This leads to more competition on the global market for poppy straw, which can increase prices if the demand is not met with adequate supply (44, 45).

## How Is the Market for Diamorphine Structured?

Over the past 20 years, according to the INCB, the licit manufacture of diamorphine worldwide averaged 700 kg annually (32). In 2019, Switzerland manufactured, consumed, and kept the largest stock of diamorphine worldwide (46).

**FIGURE 2:**
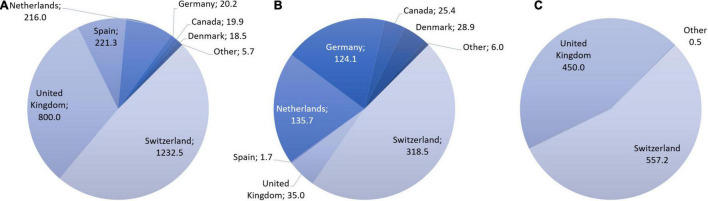
Stocks **(A)**, consumption **(B)**, and manufacture **(C)** of diamorphine in kg per country, 2019. Based on data requested from INCB.

Figure 2C shows that in 2019, a total of 1 metric ton of diamorphine was manufactured by Switzerland and the United Kingdom (557.2 kg, or 55.3%; respectively 450 kg, or 44.7%). Both countries also have the largest stocks of diamorphine in 2019 (Switzerland: 1.2 metric tons; UK 0.8 metric tons), together holding 80.2% of the stocks worldwide (Figure 2A).

In 2019, Switzerland accounted for roughly half (318.5 kg, 47.2%) of the global medical diamorphine consumption (675.4 kg). Other countries with significant diamorphine consumption are the Netherlands, Germany, the United Kingdom, Denmark, and Canada (Figure 2B). Table 1 shows that Switzerland’s consumption of diamorphine is extraordinarily high when related to its population size compared with the other countries.

**TABLE 1:** Diamorphine consumption and population size of Switzerland, Netherlands, Germany, United Kingdom (UK), Denmark, and Canada in 2019.

Country	Switzerland	Netherlands	Germany	UK	Denmark	Canada
Diamorphine consumption [in kg]*	318.5	135.7	124.1	35	28.9	25.4
% of worldwide consumption	47.2%	20.1%	18.4%	5.2%	4.3%	3.8%
Population [in Mio.]**	8.6	17.3	83.1	66.8	5.8	37.6

*INCB data 2019 **Population data from the World Bank for 2019. https://data.worldbank.org/indicator/SP.POP.TOTL (accessed on 12. January 2021).

Diaphin, is the only authorized diamorphine product in Switzerland, Germany, and Denmark, representing more than two-thirds of the world’s diamorphine consumption (3, 47, 48). Hence, DiaMo holds the monopoly in the cited countries and dominates the world market.

## Are There Legal or Regulatory Constraints Preventing the Introduction of a Diamorphine Generic in Switzerland?

Generally, the first constraint for a generic is the existence of a patent. A patent is an intellectual property right for a technical invention, such as a medicine. It allows its owner to prevent others from using the patented invention for commercial purposes for up to 20 years from the patent application (49, 50). In the case of diamorphine, there were no patents on the substance itself. Indeed, Bayer, who started commercial production of diamorphine, could not patent the substance because it was not new, as the English chemist Charles Wright had already synthesized and published a description of diamorphine in 1874 (51).

Another mechanism is document protection, also called marketing exclusivity; it prohibits competitors from relying upon and referring to data submitted to the authority by the originator company. The Swiss Therapeutic Products Act (TPA) allows 10 years of document protection for new medicines (52, 53). Before the expiry of this period, Swissmedic cannot grant a third party a marketing authorization (typically a generic authorization), which relies on the protected data (54). Swissmedic first approved the 10 g i.v. diamorphine formulation in December 2001 (3). Hence, document protection has long run out. More recently, the originator received document protection for a 5 g i.v. formulation, which will last 3 years, until 29.07.2024 (3, 55). However, this should not discourage potential competitors as the document protection only protects the 5 g formulation. Similarly, even though Diaphin has orphan drug status, it was not granted increased document protection of 15 years because it was registered before 2019 (56, 57).

Another administrative hurdle faced by companies wanting to supply diamorphine in Switzerland is applying for a narcotics license from Swissmedic. However, no exceptional license from the Federal Office of Public Health (FOPH) is needed because diamorphine is the active ingredient of an authorized medicinal product (58, 59). Swissmedic will license persons and companies, including brokers, agents, and the army pharmacy, to handle authorized medicinal products containing schedule d substances, such as diamorphine (60). In practice, it considers companies with a license to handle schedule a substances, e.g., morphine, to be also authorize to handle medicinal products containing schedule d substances (i.e., diamorphine) (61). Indeed, 321 companies had a schedule a narcotic license as of mid-December 2021 (62). All of which would, in theory, be allowed to handle diamorphine.

In summary, there is no patent protection on diamorphine, and document protection only covers 5 g Diaphin i.v. Furthermore, we find that diamorphine is subject to constraints similar to substances such as morphine. Hence, in our opinion, there are no regulatory hurdles that should discourage potential competitors from entering the Swiss market.

## How Did Switzerland Address the 2021 Supply Disruption?

According to a report on the Swiss TDP, the following actions were taken to mitigate the 2021 shortage: allocation by the manufacturer based on past orders and limitation of purchases; recommendation to switch to Diaphin IR or SR tablets or other OAT; and the postponement of the study on the nasal application of Diaphin i.v. (10). The report also stated that a new manufacturer for Diaphin i.v. had been found in the Netherlands, which resolved the 2021 supply disruption (10).

Compulsory stockpiling is a precautionary measure of import-dependent Switzerland; the stocks can be released as needed when the demand for important basic supplies, including medicines, can no longer be met on the market due to a (temporary) shortage. However, diamorphine is not subject to compulsory stockpiling by the marketing authorization holder, unlike many other opioid medicines such as morphine, fentanyl, and methadone (63). Hence, there were no compulsory stocks that could be released in response to the 2021 supply disruption.

Even though Switzerland seems to have overcome the 2021 supply disruption unscathed, it remains unprepared for a diamorphine shortage.

Compared to Switzerland, the UK has taken a more active approach to managing the risks of a potential diamorphine shortage. In 2006, the UK Office of Fair Trading issued a review that alerted the government of a monopoly situation in the UK (64). The report states: “the Government recognizes that a lack of competition may continue to have a knock-on effect on both downstream prices and the ability of competitors to enter into the market.” Nowadays, the UK has several suppliers for diamorphine (Supplementary Table 1) but still encounters shortages (65, 66). For example, the 5 and 10 mg diamorphine i.v. formulation has intermittently been in short supply since 2018 despite having two suppliers (66). In response to the supply issues, the UK National Health Service (NHS) issued recommendations and implemented medical guidelines to tackle the shortages (67). The NHS recommended switching patients permanently to morphine sulfate solution, where clinically applicable. Primary and secondary care were asked to ensure that no new patients would be started on the diamorphine i.v. strengths with unstable supply; furthermore, patients patients should not be switched to higher strengths, as there were insufficient stocks to support the increased use.

## What Do We Recommend?

Based on the information we gathered, authorities in Switzerland have done little to address the diamorphine monopoly and potential shortages. Probably because even though shortages loomed, a rupture of stock never occurred. However, the recent supply chain disruption of diamorphine and the shortage of Sevre-Long (slow-release morphine used in OAT) highlight the importance of preparing for a shortage situation (68, 69). Hence, we recommend several actions to prepare for a potential shortage, sorted by the ease of implementation.

First, authorities and relevant stakeholders should elaborate a plan describing actions to take in a shortage situation. The plan should include a national distribution key to attribute the remaining limited stocks of diamorphine among persons in TDP. This medical-ethical (triage) guideline will help physicians to navigate the shortage. Similar to the SARS-COV2 intensive care triage guidelines, there should be a national ethical, systematic and evidence-based framework to approach the patient allocation of scarce medications in general.

Secondly, diamorphine should be added to the list of compulsory stocked medicines to stabilize the supply. Considering that diamorphine has a long shelf life, this preventive measure could help alleviate short-term ruptures in the supply chain of up to 3 months.

Thirdly, long-term options to reinforce the supply chain of diamorphine should be evaluated. Indeed, the report on the Swiss TDP listed four proposals to reinforce the supply chain: optimization of the current supply by the pharmaceutical industry; decentralized manufacturing by hospital pharmacies; procurement through government; and manufacturing by the government (10).

In our view, these proposed measures are not enough to improve the situation long-term, at least as long as the monopoly remains. Decentralized manufacturing by hospital pharmacies carries logistical issues, especially for TDP centers not in close proximity to hospitals, and would likely not be cheap. Procurement by the government through a tender would likely alleviate some of the risks; however, to be successful there must be interested pharmaceutical companies. Alternatively, Switzerland could evaluate other suppliers, for example, from the UK (Supplementary Table 1). However, this would be problematic even short-term, because Swiss demand would likely extend beyond the available UK supply, as Switzerland uses almost 10 times more diamorphine annually.

Given the perceived “unattractiveness” of manufacturing diamorphine and the current monopoly, manufacturing by the Swiss army pharmacy should be evaluated. The army pharmacy is the only federal administrative unit in Switzerland that holds Swissmedic licenses to manufacture, import, wholesale trade, and export medicinal products (11). Manufacturing of diamorphine by the army pharmacy is the most radical intervention. It would require allowing the Swiss army pharmacy to compete with the pharmaceutical industry, which is unlikely to gather the needed political support (70).

Lastly, injectable hydromorphone should be evaluated as an alternative to diamorphine. The Canadian NAOMI study, a double-blind study with injectable hydromorphone and diamorphine, demonstrated that patients were unable to detect which one they received (71). The authors stated: “the fact that most patients in the hydromorphone group thought they were receiving heroin suggests that hydromorphone can effectively treat and retain opioid-dependent individuals” (71). The SALOME study provided evidence that the injectable hydromorphone was non-inferior to diamorphine for long-term opioid use disorders (72). Nevertheless, it should be kept in mind that the TDP population often has other psychiatric comorbidities (73, 74), including severe chronic anxiety, which can make them more vulnerable to changes. Hence, switching patients from diamorphine to hydromorphone, in case of a shortage, could destabilize a person in treatment. Moreover, reimbursement could be problematic as hydromorphone would be used off-label, and the treatment with hydromorphone instead of diamorphine would be likely several times more expensive. To our knowledge, there has been no large-scale use of hydromorphone for the treatment of opioid use disorders in Switzerland.

In summary, diamorphine should be added to the list of compulsory stocks, and triage guidelines should be elaborated for the allocation of scarce medicines. The potential of the Swiss army pharmacy to manufacture diamorphine should be evaluated if there are not enough pharmaceutical companies interested in bringing other diamorphine products on the market.

## Conclusion

In conclusion, Switzerland has so far been lucky in that it has not suffered a shortage of diamorphine; nevertheless, the unstable supply was evidenced recently by reports in Switzerland and Germany. Measures that prevent a shortage in the future and precise planning for a shortage situation must be implemented. TDP is a valuable and successful public health program, and an insufficient supply of diamorphine would affect persons in treatment but also the society as a whole. Switzerland surely does not want to go back to the extreme and very public desperation of the open drug scenes.

## Data Availability Statement

The dataset of diamorphine manufacturing, stocks, and consumption in 2019, used in this article, can be requested from the International Narcotics Control Board (INCB). Further enquiries can be directed to the corresponding author.

## Author Contributions

CS-K: investigation, data curation, and writing—original draft preparation. CS-K, C-AB, VJ, and OS: writing—review and editing. VJ and OS: supervision and project administration. VJ, C-AB, and OS: funding acquisition. All authors have read and agreed to the published version of the manuscript.

## Conflict of Interest

The authors declare that the research was conducted in the absence of any commercial or financial relationships that could be construed as a potential conflict of interest.

## Publisher’s Note

All claims expressed in this article are solely those of the authors and do not necessarily represent those of their affiliated organizations, or those of the publisher, the editors and the reviewers. Any product that may be evaluated in this article, or claim that may be made by its manufacturer, is not guaranteed or endorsed by the publisher.
