# Supplementary material for: Switzerland’s Dependence on a Diamorphine Monopoly
Source: Front Psychiatry. 2022 May 9;13:882299. doi: 10.3389/fpsyt.2022.882299 (PMC9125182; doi:10.3389/fpsyt.2022.882299)
Supplement: Supplementary file 1 [file Table_1.DOCX]

Supplementary Material

Supplementary Table 1. Comparison of diamorphine prescription (TDP) programs in Switzerland, Germany, and United Kingdom. OAT= opioid agonist treatment. *Data from UK NHS dm+d browser: https://services.nhsbsa.nhs.uk/dmd-browser/search (accessed on 20. December 2021) **UK drug tariff (December 2021): 5 ampoules Diamorphine 500mg = £187

|  | **Switzerland** | **Germany** | **United Kingdom** |
| --- | --- | --- | --- |
| **Diamorphine consumption (2019)** | 319kg | 124kg | 35kg |
| **# persons in TDP (2019)**  **% TDP of total OAT** | 1’663  (9.2% of OAT) | 877  (1.1% of OAT) | 280  (0.2% of OAT) |
| **Market authorization holders** | DiaMo | DiaMo | Wockhardt UK Ltd, A A H Pharmaceuticals Ltd, Alliance Healthcare (Distribution) Ltd, Accord Healthcare Ltd* |
| **i.v. price / 10g** | CHF 205 | €260 | £748** |
